# Transgene Expression Is Associated with Copy Number and Cytomegalovirus Promoter Methylation in Transgenic Pigs

**DOI:** 10.1371/journal.pone.0006679

**Published:** 2009-08-18

**Authors:** Qingran Kong, Meiling Wu, Yanjun Huan, Li Zhang, Haiyan Liu, Gerelchimeg Bou, Yibo Luo, Yanshuang Mu, Zhonghua Liu

**Affiliations:** 1 College of life science, Northeast Agricultural University of China, Harbin, People's Republic of China; 2 College of Medicine and Pharmaceutics, Ocean University of China, Qingdao, People's Republic of China; Brunel University, United Kingdom

## Abstract

Transgenic animals have been used for years to study gene function, produce important proteins, and generate models for the study of human diseases. However, inheritance and expression instability of the transgene in transgenic animals is a major limitation. Copy number and promoter methylation are known to regulate gene expression, but no report has systematically examined their effect on transgene expression. In the study, we generated two transgenic pigs by somatic cell nuclear transfer (SCNT) that express green fluorescent protein (GFP) driven by cytomegalovirus (CMV). Absolute quantitative real-time PCR and bisulfite sequencing were performed to determine transgene copy number and promoter methylation level. The correlation of transgene expression with copy number and promoter methylation was analyzed in individual development, fibroblast cells, various tissues, and offspring of the transgenic pigs. Our results demonstrate that transgene expression is associated with copy number and CMV promoter methylation in transgenic pigs.

## Introduction

Transgenic animals are a powerful tool in the fields of experimental and applied biology. These animals allow study into the function and regulation of genes in vivo, the production of important pharmaceutical proteins, and the creation of pathologic models for human disease therapy [1]. Recent progress in animal cloning has provided an attractive alternative to improve transgenic efficiency, through the combination of transfection and somatic cell nuclear transfer (SCNT).

To date, the cloning of pigs has been used successfully to produce transgenic animals expressing enhance green fluorescence protein (eGFP) [2] and omega-3 fatty acids [3], and an α-1,3-galactosyltransferase deficient pig, which has the potential to be used as an organ donor for xenotransplantation [4]. The production of genetically modified pigs by nuclear transfer has progressed from basic research to practical use. Despite this impressive and growing success, transgenesis still suffers from many limitations. Numerous experiments have shown that the inheritance and expression of the transgene in transgenic animals is predictable only to a limited extent [5]–[8]. In most cases, transgene expression levels in transfected cells often decline with time [6]. Furthermore, the level of transgene expression appears to correlate inversely with time [9], and the majority of transgenic animals cannot stably pass the transgene to their offspring. Therefore, it is difficult to select founder transgenic animals to establish a line of transgenic animals [7]. Recently, several reports have demonstrated that induced pluripotent stem cells (iPS) may possess mechanisms to lower the expression level of the four factors (Oct3/4, Sox2, c-Myc, and Klf4) and make them expression silencing after full-reprogramming [10]–[12].

Mechanisms causing this phenomenon of transgene instability are poorly understood. Generally, transgene copy number and DNA methylation status can influence transgene expression [13]. These are often considered as main factors resulting in incomplete and complete silencing of transgene expression [14]. In most cases, multiple copies of the transgene, arrayed in a head-to-tail manner, are randomly integrated in the host genome, which may cause transcriptional interference that represses expression [15]–[17]. As long as the transgene is expressed appropriately, calculating the copy number is not usually performed. DNA methylation is the strongest candidate for expression silencing, because it can lead to transcriptional inactivity of certain genes, may be stably inherited through mitosis, and may be transmitted to subsequent generations [18]–[20]. In particular, promoter methylation has been associated with transgene silencing *in vitro* and *in vivo* [21]–[23].

A more clear understanding of the factors influencing transgene expression would improve the production of transgenic animals. In order to test the relationship between transgene expression and copy number or promoter methylation, we generated two GFP transgenic pigs by SCNT and analyzed GFP expression, copy number and CMV methylation in regards to individual development, fibroblast cells, various tissues, and the offspring of the transgenic pigs. Our results suggest that transgene expression is regulated by methylation of the promoter and by transgene copy number.

## Results

### Generation of transgenic pig

A total of 1978 reconstructed embryos were transferred to ten recipients. Three recipients became pregnant and four founder GFP-positive transgenic pigs were born at full term. Finally, two female pigs, named K25-2 and K25-3, survived to maturity (the others died at birth). After mating with non-transgenic pigs, K25-2 produced seven F1 positive pigs from two litters, but K25-3 died during delivery. Variable factors may decrease the success rate of producing transgenic animals, and an additional difficulty in pigs requires that at least four good embryos are needed to induce and maintain pregnancy [3]. In the present study, the overall efficiency of transgenic pig production was 0.69%.

### Expression of GFP

**Figure 1:**
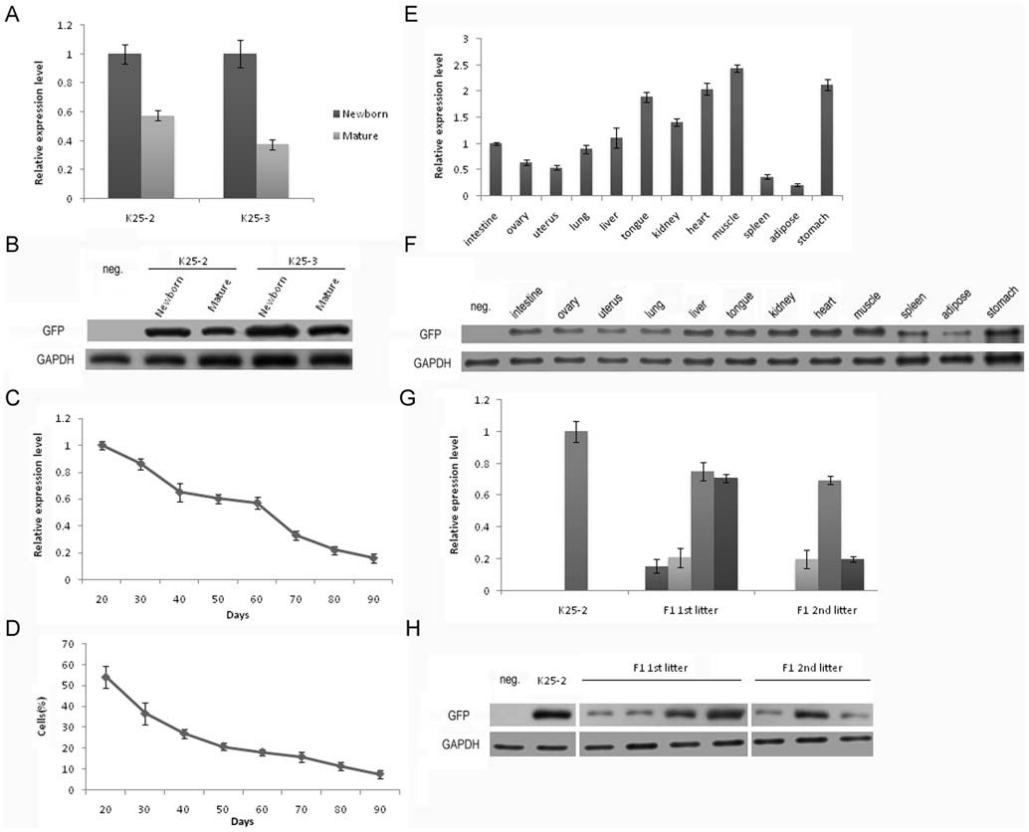
Expression of GFP. (A) Relative real-time RT-PCR analysis of GFP mRNA expression from newborn to maturity in founder transgenic pigs. The decline was significant in both K25-2 and K25-3 (p<0.001); (B) Western blots analysis of GFP protein from newborn to maturity in founder transgenic pigs; (C) Relative real-time RT-PCR analysis of GFP mRNA expression in transgenic fibroblast cells. The decline of mRNA from 20 to 90 days was significant (p<0.001); (D) Flow cytometry analysis of the percentage of phenotype positive cells. The decline of percentage of positive cells from 20 to 90 days was also significant (p<0.001); (E) Relative real-time RT-PCR analysis of GFP mRNA expression in various tissues of the transgenic pig. Variegation of GFP mRNA expression was shown in different tissues (p<0.001); (F) Western blots analysis of GFP protein in various tissues; (G) Relative real-time RT-PCR analysis of GFP mRNA expression in offspring transgenic pigs. The decline from founder to offspring was significant (p<0.001); (H) Western blots analysis of GFP protein in offspring transgenic pigs. Error bars denote standard deviations. Neg., non-transgenic pig.

We analyzed the change in GFP expression in relation to aging in ear tissues of transgenic pigs. A significant decline in the mRNA level was observed from newborn to maturity in both K25-2 and K25-3 (p<0.001). The mRNA level decreased about 1-fold and 3-fold in K25-2 and K25-3, respectively (Fig. 1A). These results were consistent with results from Western blot analysis (Fig. 1B). These data indicate that the transgene expression level decreases with aging *in vivo*.

In order to examine whether the expression of the transgene also declined *in vitro*, fibroblast cells from K25-3 were cultured *in vitro* up to 90 days were analyzed for transgene expression. A significant decline (more than 5-fold) in mRNA levels was observed from 20 to 90 days in culture (p<0.001) (Fig. 1C). Flow cytometry analysis also showed a significant decline (almost 8-fold) in the percentage of cells that expressed GFP (p<0.001) (Fig. 1D). A decrease in fluorescence intensity from 20 to 90 days was also found by flow cytometry analysis (data not shown). Previous reports have demonstrated that transgene silencing in transgenic cells may occur through a decline in expression levels rather than in the proportion of expressing cells [6], [17], [24], but the present data do not clarify this point, and a decrease in the percentage of positive cells was evident.

**Figure 2:**
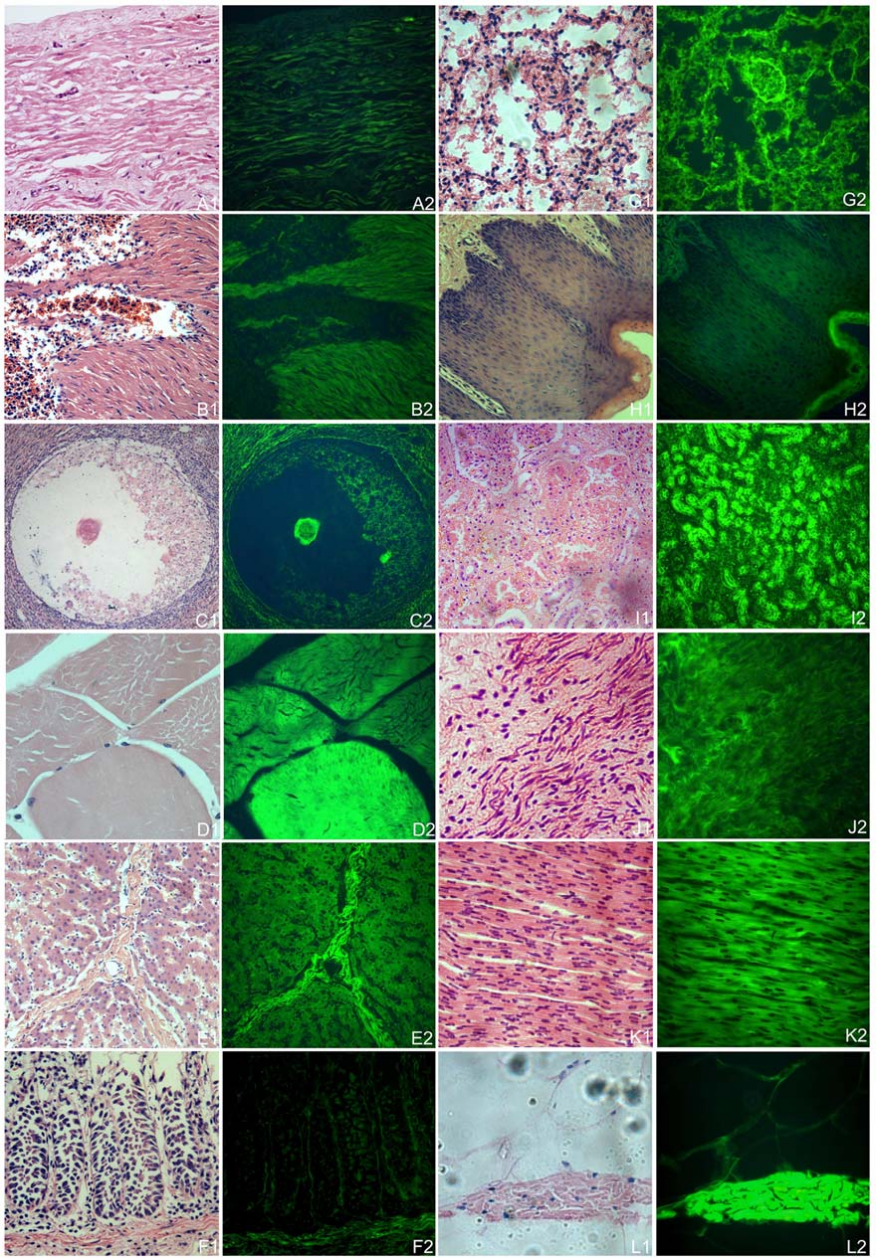
Variegation of GFP expression in various tissues of the transgenic pig. A to L: different tissues, namely uterus, spleen, ovary, muscle, liver, intestine, lung, tongue, kidney, stomach, heart and adipose, were visualized by HE staining. A1 to L1: tissues under normal light. A2 to L2: tissues under UV light.

GFP expression was detected in different tissues of K25-3, namely intestine, ovary, uterus, lung, liver, tongue, kidney, heart, muscle, spleen, adipose and stomach. However, significant difference was found in the mRNA levels observed in these tissues (p<0.001). In tongue, heart, muscle and stomach, mRNA levels were almost 20-fold higher compared to that in spleen, adipose, ovary and uterus (Fig. 1E). The difference was confirmed by Western blot analysis (Fig. 1F). To examine the GFP expression pattern directly, different tissues were observed under UV light (Fig. 2), and a variegation of expression was observed. These results are consistent with previous studies in that transgene expression in transgenic animals may be different between tissues [25], [26].

GFP expression was detected in offspring of K25-2, and was varied. The mRNA expression in offspring was significantly lower (more than 5-fold) compared to that in the founder (p<0.001) (Fig. 1G). Western blot analysis also showed a decline between K25-2 and its offspring (Fig. 1H). These findings suggest that the expression of the transgene in transgenic animals does not stably pass to their offspring.

### Copy number of GFP

**Figure 3:**
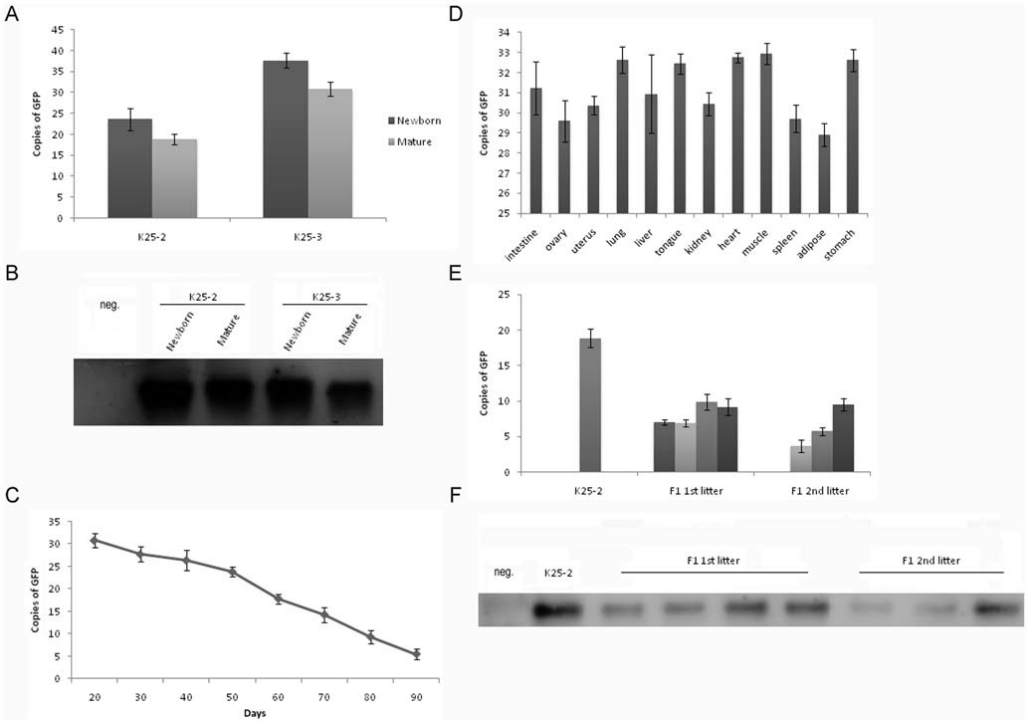
Copy number of GFP. (A) Absolute quantitative real-time PCR analysis of GFP copy number from newborn to maturity in founder transgenic pigs. There was no statistically significant decline in K25-2 (p = 0.099), but the decline in K25-3 was significant (p = 0.016); (B) Southern blots analysis of GFP copy number in newborn and mature transgenic pigs; (C) Absolute quantitative real-time PCR analysis of GFP copy number in transgenic fibroblast cells. Copy number of GFP declined in cells over time in culture. The decline was significant (p<0.001); (D) Variegation of GFP copy number in various tissues of transgenic pig. Variegation of GFP copies was shown in different tissues (p = 0.059); (E) Absolute quantitative real-time PCR analysis of GFP copy number in offspring transgenic pigs. The decline from founder to offspring was significant (p<0.001); (F) Southern blots analysis of GFP copy number in offspring transgenic pigs. Error bars denote standard deviations. Neg., non-transgenic pig.

In order to determine the correlation of transgene expression with copy number, we examined the GFP copy number in ears of newborn and mature transgenic pigs. A decline in copy number was found by absolute quantitative real-time PCR (Fig. 3A) and Southern blot analysis (Fig. 3B). Although a significant decline was not detected in K25-2 (p = 0.099), the decline in K25-3 was significant (p = 0.016). A more than 4-copy drop was observed in K25-2, and a more than 6-copy drop was observed in K25-3 (table 1). Transgenic animals show a difference in transgene copy number [27], [28], but a decline in the copy number with aging is not a common observation.

**Table 1:** GFP copies in newborn and mature founder transgenic pigs.

Pig	Term	No. of Copies
**K25-2**	Newborn	23.62^a^±2.59
	Mature	18.87^a^±1.34
**K25-3**	Newborn	37.59^b^±1.79
	Mature	30.85^c^±1.77

Note: Different superscripts indicate statistical difference (P<0.05).

We also analyzed the GFP copy number in transgenic fibroblast cells cultured in vitro for up to 90 days. The data showed that the GFP copy number declined significantly (p<0.001) and the copy number decreased almost 6-fold from 20 to 90 days (Fig. 3C). These results are consistent with the conclusion that transgene copies may be lost in transgenic cells over time *in vitro* [6]. In addition, we observed a significant correlation between GFP expression levels and copy number in transgenic fibroblast cells (r = 0.965, p<0.001). These results suggest that the decline of transgene expression may be due to the loss of copies.

The GFP copy number in various tissues of K25-3 was also tested (Fig. 3D). Although the copy number in different tissues was varied, the difference was not significant (p = 0.059) and not clearly shown by Southern blots (data not shown). In muscle, heart and lung, the GFP copy number was about 3 copies more than that in adipose, ovary and spleen. In addition, the correlation between GFP expression and copy number in different tissues was significant (r = 0.851, p<0.001). In our earlier report, we demonstrated a mosaic genotype in a transgenic pig that died at birth. In that animal, we did not observe GFP sequence in muscle by PCR analysis [29], but, in the present study, this phenomenon was not observed.

Transgene loss during passage is common [27], [30]. In present study, the loss of GFP copies during passage of K25-2 was significant (p<0.001), and the GFP copy number in different offspring varied (Fig. 3E). Moreover, the observed loss of GFP copies was 15 copies at most and 9 copies at least (Table 2). Southern blots confirmed the decline (Fig. 3F). The correlation of GFP expression level with copy number was significant in this case (r = 0.864, p = 0.006). These results suggest that transgenic animals may transmit the transgene to their offspring only to a limited extent, and this loss of transgene copies is responsible for the expression decline.

**Table 2:** Copy number of GFP in K25-2 and its offspring.

Generations	Litters	Pigs	No. of Copies
**F0**	—	K25-2	18.87^a^±1.34
**F1**	First	1	7.01^cd^±0.34
		2	6.86^cd^±0.52
		3	9.89^b^±1.09
		4	9.19^bc^±1.20
	Second	1	3.67^e^±0.59
		2	5.71^de^±0.85
		3	9.54^bc^±0.84

Note: Different superscripts indicate statistical difference (P<0.05).

### Methylation status of CMV promoter

Transgene expression may be regulated by copy number and repressed by DNA methylation. Therefore, we examined the methylation status of a 278-bp region of the CMV promoter containing one CpG island with 14 CpG sites. The bisulfite sequencing method is able to reveal the methylation status of all the cytosine residues in a DNA region of interest [31].

**Figure 4:**
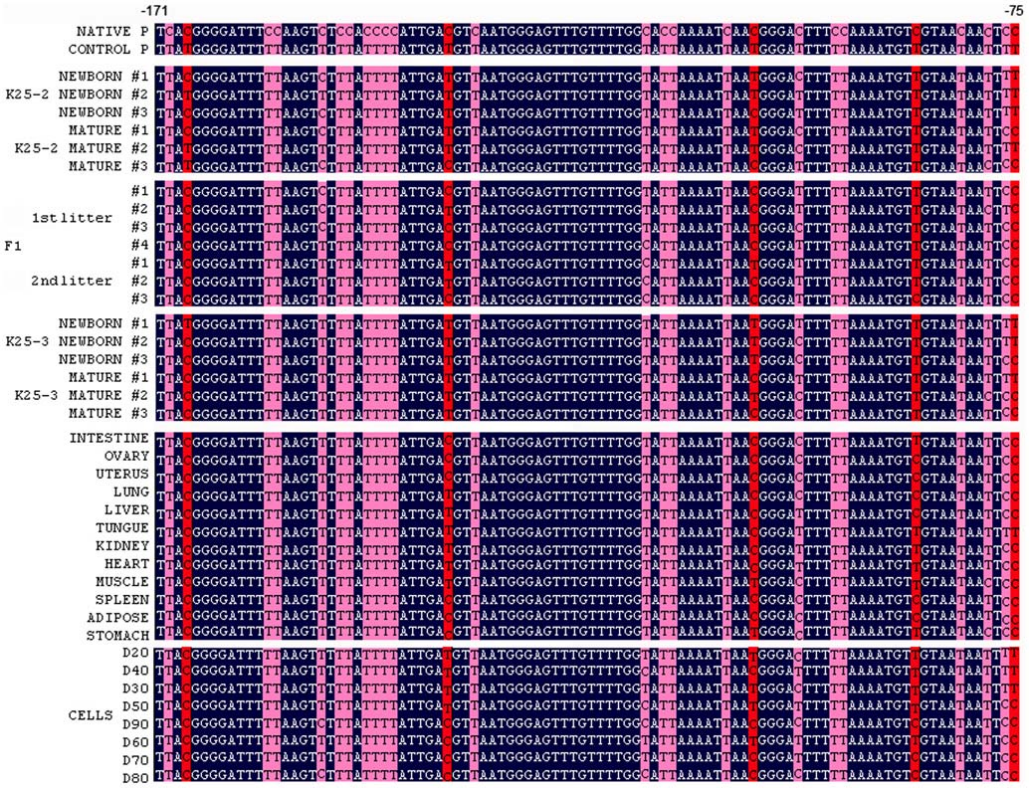
Bisulfite sequence data for a representative region of CMV promoter. Native P is the native sequence of the CMV promoter. The sequences from clones obtained after bisulfite treatment of DNA samples were aligned to the native sequence. For example, in control P cytosine residues were not methylated and therefore converted to thymidine by the bisulfite treatment. CpG and non-CpG cytosines were highlighted on a red and pink background, respectively.

The bisulfite sequence data for a representative region of CMV promoter is shown in Fig. 4). On average, 99% of the cytosine residues in the pEGFP-C1 plasmid were converted to thymidine, indicating that the bisulfite conversion reaction on other samples was at least 99% efficient. The converted or unconverted cytosines at CpG sites (on a red background) indicate unmethylated or methylated. Interestingly, there were some unconverted cytosines at non-CpG sites (on a pink background), indicating cytosines that might be methylated. Extensive non-CpG methylation of the CMV promoter has been reported and associated with transgene silencing [22]. However, in this study, these cytosines were not included methylation level analysis because we did not know the mechanism of non-CpG methylation.

**Figure 5:**
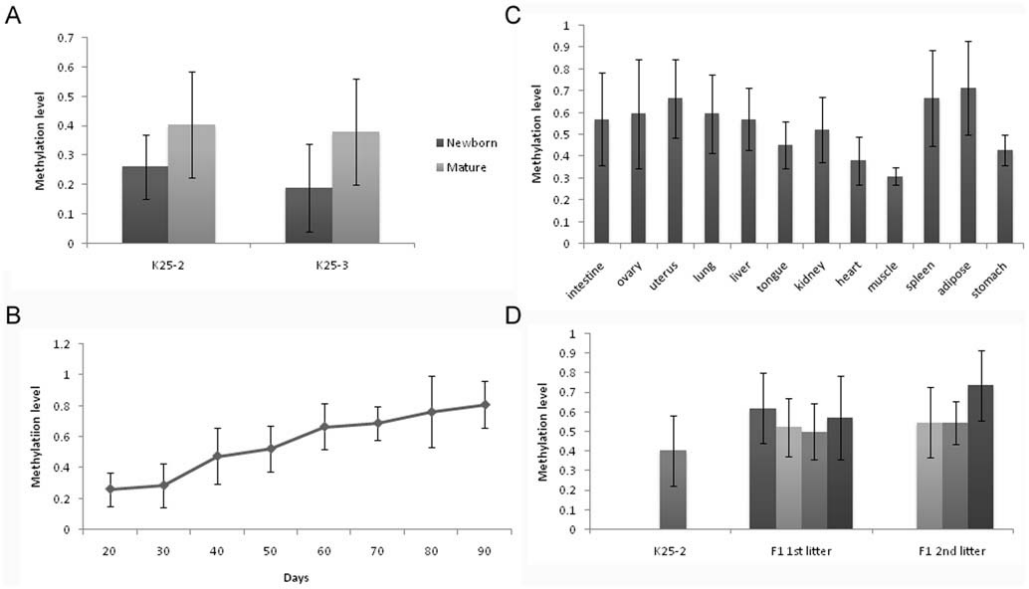
Methylation of CMV promoter. (A) Level of CMV methylation increased in founder transgenic pigs from newborn to maturity. The increases in CMV methylation level from 26% to 40% (p = 1.000) and from 19% to 38% (p = 0.799) were observed in K25-2 and K25-3, respectively; (B) Level of CMV methylation increased in transgenic fibroblast cells over time in culture. The increase in CMV methylation level from 20 to 90 days was more than 3-fold from 26% to 81% (p = 0.053); (C) Variegation of CMV methylation in various tissues of transgenic pig. Hypermethylation and hypomethylation levels of CMV methylation were found in different tissues, but the difference was not significant (p = 0.153); (D) Level of CMV methylation in offspring transgenic pigs. The increase in CMV methylation level from 40% to 58% on average of offspring was detected (p = 0.537). Error bars denote standard deviations.

We detected CMV methylation levels in ears of newborn and mature transgenic pigs, and observed an increase from 26% to 40% (p = 1.000) and from 19% to 38% (p = 0.799) in K25-2 and K25-3, respectively (Fig. 5A).

In the analysis of CMV methylation in transgenic fibroblast cells cultured from 20 to 90 days (Fig. 5B), we found that the level increased more than 3-fold from 26% to 81% (p = 0.053), and the increase in CMV methylation was conversely correlated with GFP expression (r = −0.967, p<0.001). These results suggest that the methylation level of the transgene promoter appears to increase *in vivo* or *in vitro*, and to repress transgene expression.

A variegation of CMV methylation in different tissues of K25-3 was detected (Fig. 5C). There was a hypermethylation level in uterus (67%), spleen (67%) and adipose (71%), and a hypomethylation level in heart (38%) and muscle (30%). However, the difference in the methylation levels in different tissues was not significant (p = 0.153). The correlation of GFP expression with CMV methylation in different tissues was significant (r = −0.982, p<0.001), indicating that transgene expression is associated with promoter methylation in transgenic animal tissues, which is consistent with a recent report [23].

A change in CMV methylation during passage was also observed between K25-2 and its offspring (Fig. 5D). The increase in CMV methylation levels between founder and offspring, from 40% to 58% on average of offspring, was not significant (p = 0.537), and there was no significant correlation of GFP expression with CMV methylation (r = −0.682, p = 0.063). Though a wide range of methylation levels from 50% to 74% among offspring was observed, the difference was not significant (p = 0.615). The complex inheritance of transgene promoter methylation seems to suggest randomly incomplete erasure and reset.

In these cases, we did not find significant differences in the methylation level, and the results observed may be related to cellular mosaicism and environment influences on methylation status [20], [32].

### Effect of copy number and promoter methylation on transgene expression

In order to determine which factor, copy number or promoter methylation, is more significant in the determination of transgene expression, a multivariate linear regression model was employed. The linear regression fit has been shown to provide a reasonably close approximation to estimate the correlation of gene expression with copy number and DNA methylation [33], [34]. In these models below, E, M and C represents GFP expression level, CMV methylation level and GFP copy number, respectively, and β_M_ and β_C_ represents the deterministic force of CMV methylation and GFP copy number, respectively.

In analysis of transgenic fibroblast cells over a long time course in culture, the model was: E = −5.319M+0.065C+2.063 (r = 0.984, p<0.001) and β_M_ = −0.88, β_C_ = 0.124. We also analyzed the correlation in different transgenic tissues, and the model was: E = −0.74M+0.015C+0.667 (r = 0.977, p<0.001) and β_M_ = −0.517, β_C_ = 0.472. These results indicate that although both copy number and promoter methylation can regulate transgene expression levels in fibroblast cells and tissues, promoter methylation appears to be more significant in affecting transgene expression compared to copy number. The model in the passage of the transgenic pig was: E = −0.354M+0.069C−0.319 (r = 0.865, p = 0.032) and β_M_ = −0.102, β_C_ = 0.947, which suggests that copy number is superior to promoter methylation in transgene expression regulation.

## Discussion

To our knowledge, this is the first study to systematically analyze the effect of copy number and promoter methylation on transgene expression in domestic animals. Our results suggest that transgene expression level is associated with copy number and CMV promoter methylation status in transgenic pigs.

In random transfection, the transgene usually integrates into the host genome in a tandem manner. High copy number tandem integration is thought to lead to transgene silencing [17], [35], however, in the present study, the correlation of transgene expression level with copy number was positive (β_C_>0). This difference may be caused by a position effect, and further experiments are needed to clarify the exact mechanism. CMV is very strong promoter *in vitro* [36]. Several reports have described the CMV promoter as being silent *in vivo*, and described a role of DNA methylation in silencing of the CMV promoter [17], [37]–[39]. A negative correlation between methylation of the promoter and gene expression has been documented previously [40], [41], and was confirmed in our study (β_M_<0).

The decline of transgene expression with time may contribute to the increase in promoter methylation rather than the loss of copy number. Previous reports have shown that transgene expression level declines with time *in vivo* and *in vitro* [6], [9]. This is similar with the results observed here, and, in addition, both a loss of copy number and increase in promoter methylation were observed. The loss of a transgene integrated into the host genome has been observed in K562 cells over a long time course in vitro [6]. However, we demonstrated the decline of transgene copy number with aging in transgenic animals. The transgene copy number decrease can be understood in two points below: firstly, it is position dependent. A few reports described there were some sites where transgene was inclined to integrate by random transfection, such as LINE elements [6], [27]. We believe the random transfection may not result in random integration instead in some hot integration sites and these hot integration sites may have the common character easy for transgene to integrate, but, we propose the hypothesis, it may be also easy for transgene to lose; secondly, it is sequence dependent. The transgene we used is eGFP which is heterogeneous from a kind of medusa, and we propose there may be a sequence dependent mechanism to immune-mediated destruction of transgene, as in the deletion of endogenous virus [42], [43]. An increase in transgene promoter methylation was demonstrated during individual development of transgenic pigs and transgenic fibroblast cells. This observation contradicts previous conclusions that established methylation patterns can be maintained and stably transmitted during mitosis [18], [20]. A gradual modification of methylation has been observed during individual development of cloned pigs, and it is important to correct the aberrant expression of imprinted genes in cloned embryos and offspring [44], [45]. The mechanism of increase in transgene promoter methylation may be related to a defense system targeted against the transgene [46], [47]. In this case, it seems that the decline of transgene expression may be caused by both the loss of copy number and an increase in promoter methylation, but, according to the β-value, the latter likely plays a more important role.

The variegation of transgene expression in different tissues of transgenic animals is more closely correlated with promoter methylation than copy number. The CMV promoter exhibits various expression profiles. Villuendas et al. (2001) [48] and Van den Pol et al. (1998) [35] reported that the CMV promoter is active in neurons, testis and certain other tissues of transgenic mice, but Yang PH et al. (2008) [28] did not detect CMV activity in heart, liver, spleen, lung, kidney, skin and muscle of transgenic cattle. In our study, GFP expression was detected in all the tissues examined, but the expression levels were different between tissues. Varied transgene copies and promoter methylation were found in tissues. A difference in DNA methylation among tissues is well known, and the methylation status of an identical site in different tissues may also be different [49]. Thus, the differential promoter methylation status of the transgene among several tissues is consistent to the previous observations. However, the mechanism leading to the variegation of transgene copies is not clear. Transgene in different tissues of transgenic animals generated by pronuclear microinjection observed a mosaic pattern has been reported [50], [51]. But in the case of SCNT animals, all the subsequent offspring and the cells within them originate from a single donor cell status which basically reflects a single genotype and its epigenetic status. Different cell division rates in different tissues may lead to a difference in the extent of loss of transgene. Nevertheless, according to the data reported here, different transgene copy numbers and the promoter methylation status in different tissues may be responsible for the expression variegation.

The decline of transgene expression in offspring seems to result in a loss of the transgene during passage. Founder transgenic pigs mated with non-transgenic pigs may reduce the copy number, consistent with previous works [27], [52]. DNA methylation associated with gene silencing is considered to be inherited during mitosis, but cleared during meiosis, enabling the genome to return to the totipotent state. Classic models of *de novo* methylation describe erasure through two germlines, and then a resetting. However, some reports have suggested that DNA methylation is not completely erased, but inherited [53]–[55]. In this study, we could not observe clearly the inheritance of the methylation status, but an increase in promoter methylation level was obvious in the offspring. Kearn M et al. (2000) [20] reported that a transgene inherited from the mother is completely silenced in some offspring, but, in all the genotype positive transgenic pigs we obtained, the expression of the transgene transmitted by the mother was detected. However, an obvious decline was observed, and, according to multivariate linear regression analysis, the decline is attributed to the loss of transgene copy number.

In the study, we found 1) decline of transgene copy number and increase of promoter methylation level with time *in vivo* or *in vitro*, and during passage, 2) the variegation pattern of transgene copy number and promoter methylation status in various tissues, and 3) all of these are associated with the changes of transgene expression. It is known that the correlation of promoter methylation and copy number with gene expression is not very tight, and position effect, histone modifications or other epigenetic factors can also influence transgene expression [20], [23]. However, in conclusion, our results at least demonstrate that transgene copy number and promoter methylation are responsible for the regulation of transgene expression in transgenic pigs.

## Materials and Methods

### Ethics statement

All the treatments of animals in this research followed by the guideline of Northeast Agriculture University and were approved by the committee. All animals (pigs) involved in this research were raised and breed followed the guideline of Animal Husbandry Department of Heilongjiang, P.R.China.

### Establishment of GFP transgenic pigs and passage

Fibroblast cells derived from E32 fetuses were transfected by liposome-mediated plasmid pEGFP-C1 (Clonech) containing eGFP driven by the CMV promoter, which was based on a random insertion of nonhomologous DNA vector into host genome. After G418 selection, surviving cells were used as a nuclear donor, and nuclear transfer was preformed as described [29]. After sexual maturity, founder transgenic pigs were mated with non-transgenic pigs to passage. Positive transgenic pigs were identified by PCR using primers P1 and P2. The sequences of the primers were 5′-TGAACCGCATCGAGCTGAAGGG-3′ (forward) and 5′-TCCAGCAGGACCATGTGATCGC-3′ (reverse), and PCR generated a 308-bp product. All DNA samples were extracted using the Universal Genomic DNA Extraction Kit Ver.3.0 (TaKaRa) according to the manufacturer's instructions.

### Southern blots

Each DNA sample was cleaved with EcoR I and Nhe I(TaKaRa), which can digest the pig genome efficiently and form a 800 bp fragment. The hybridization probe used to detect the GFP transcription unit DNA (753 bp) was synthesized by PCR using primers P3 and P5 and labeled by DIG Oligonucleotide 3′-End Labeling Kit (Roche). The sequences of the primers were 5′-GAGCAAGGGCGAGGAGCTGTTCA-3′ (forward) and 5′-TGCAGAATTCGAAGCTTGAGC-3′ (reverse).

### Real-time PCR analysis

#### Real-time PCR procedure

Real-time PCR was performed using SYBR Premix Ex Taq™ (TaKaRa) and the 7300 Real-Time PCR System (Applied Biosystems), with the following parameters: 95°C for 10 sec, followed by 40 two steps cycles at 95°C for 5 sec and at 60°C for 31 sec. For RT-PCR, total RNAs were extracted from each sample using the PureLink™ Micro-to-Midi system (Invitrogen) according to the manufacturer's instructions, and reverse transcription was to generate cDNAs using PrimeScript™ RT Reagent Kit (TaKaRa). Primers for the GFP gene were 5′-TGAACCGCATCGAGCTGAAGGG-3′ (forward) and 5′-ACCTTGATGCCGTTCTTCTGCTTG-3′ (reverse). For absolute quantitative PCR, the TFRC gene was used as a reference gene, and the primers were 5′-GAGACAGAAACTTTCGAAGC-3′ (forward) and 5′-GAAGTCTGTGGTATCCAATCC-3′(reverse). In relative quantitative RT-PCR, the β-actin gene was used as a reference gene, and the primers were 5′-AGATCGTGCGGGACATCAAG-3′ (forward) and 5′-GCGGCAGTGGCCATCTC-3′ (reverse). The sizes of the amplification products were 110 bp for the GFP gene, 81 bp for the TFRC gene and 93 bp for the β-actin gene. For each DNA and cDNA sample, both target and reference genes were always amplified independently on the same plate and in the same experimental run in triplicate. The melting curve analysis showed that all reactions were free of primer–dimers or other non-specific products (data not shown). C_t_ value was calculated by the Sequence Detection System software (Applied Biosystems). In relative quantitative RT-PCR, the amount of target normalized to reference was calculated by: 2^−ΔΔCt^.

### Establishment of the absolute quantitative standard curve

In order to examine the GFP copy number, generation of the absolute quantitative standard curve was necessary. First, we prepared a series of standard samples containing 1, 2, 4, 8, 16 copies of the GFP gene respectively, by mixing the wild type genome of an E32 pig with plasmid pEGFP-C1. To make a standard sample contain one copy of the GFP gene, the quality of plasmid mixed with genomic DNA was: 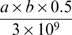
ng (“a” represents the size of plasmid). According to this principle, the standard samples containing 1, 2, 4, 8, and 16 copies of the GFP gene were prepared. The absolute quantitative standard curve was drawn by plotting △C_t_ (△C_t_ =  C_tGFP_ − C_tTFRC_) against the log of GFP gene copies of corresponding standard samples. The parameters of the standard curve was: log_2_N = −0.9354△C_t_+3.4116 (R^2^ = 0.9974, p<0.001).

### Bisulfite sequencing

Bisulfite modification was performed on 0.3 ug of DNA from each sample using the EZ DNA Methylation-Gold™ Kit (Zymo research), according to the instruction manual. PCR primers to amplify the CMV were designed by MethPrimer software on line (http://www.urogene.org/methprimer/), which was also used to predict CpG islands and CpG sites in the sequence. The following PCR primers could efficiently and specifically amplify a 278-bp region containing one CpG island with 14 CpG sites: gps: 5′TGATTTTATGGGATTTTTTTATTTG3′ (forward) and gpa: 5′ATTCACTAAACCAACTCTACTTATATAAAC3′ (reverse). None of these bisulfite dependent residues lie within the first 6 bp of the 3′ end of the primers, so if some of these were not bisulfite converted this should have a limited effect upon primer efficiency. The amplification of bisulfited-modified DNA was performed using Hot start Taq™ polymerase (TaKaRa), with the following conditions: 94°C for 5 min, followed by 40 three steps cycles at 94°C for 30 sec, 56°C for 30 sec and at 72°C for 1 min. The PCR products were separated on 1% agarose gels and purified, followed by sequencing (Invitrogen). The presence of a cytosine residue after bisulfite treatment shows that the cytosine residue was protected by methylation from bisulfite modification. For each DNA sample, the number of cytosine residues that remained as a cytosine was counted, and converted to a percentage of the 14 CpG cytosines present in the 278-bp region of the CMV that was analyzed. For the control, the pEGFP-C1 plasmid was treated and analyzed. At least five clones were sequenced and analyzed for each sample.

### Western blots

For Western blot analysis, total proteins were isolated from different tissues of K25-3 and the ear of K25-2 and its offspring by homogenization in lysis buffer (50 mM Tris-HCl, pH 7.5, 150 mM NaCl, 1% Triton X-100, 0.25% sodium deoxycholate, and complete protease inhibitor cocktail (Roche)). The concentration of proteins was measured by Bradford reagent (Sigma), separated on 10% SDS-PAGE gels and transferred to Immobilon-P membranes (Millipore). After blocking in 5% low-fat milk in PBST (0.1% Tween 20 in PBS) for 1 h, the membranes were incubated with GFP antibody (1∶500, Santa Cruz Biotechnology) or rabbit anti-Gapdh polyclonal antibody (1∶2000, Santa Cruz Biotechnology) overnight at 4°C. After washing in PBST, the membranes were incubated in goat anti-rabbit antibody conjugated with horseradish peroxidase (1∶5000) for 1 h, followed by three washes in PBST. The signals were detected by ECL Chemiluminescent kit (Amersham Pharmacia Biotech, Arlington Heights).

### Flow cytometry analysis

Fibroblast cells isolated from the ear of newborn K25-3 were cultured and proliferated in DMEM+20% FBS (Gibco). The fluorescence intensities of fibroblast cells over the time were analyzed in a FACS Calibur (Becton-Dickinson). The argon laser was tuned at 488 nm, and fluorescent cells were evaluated with a 525 nm band-pass filter. To set the parameters for flow cytometry analysis, non-transfected fibroblast cells were used as a negative control.

### Statistical analysis

Statistical analysis was preformed using SPSS 13.0 for MicroSoft™ Windows. Data are shown as mean±SD. One-way ANOVA was used to assess differences between groups. Duncan method was employed for pairwise comparison and followed by Bonferroni correction. Pearson correlation coefficient (PCC) and multivariate linear regression analysis were performed to determine independent associations between GFP expression levels and the variables of interest. P<0.05 (two-tailed) was considered statistically significant.
